# Correction: Excess mortality across countries in the Western World since the COVID-19 pandemic: ‘Our World in Data’ estimates of January 2020 to December 2022

**DOI:** 10.1136/bmjph-2023-000282corr1

**Published:** 2026-08-11

Mostert S, Hoogland M, Huibers M, Kaspers G. Excess mortality across countries in the Western World since the COVID-19 pandemic: ‘Our World in Data’ estimates of January 2020 to December 2022. *BMJ Pub Health* 2024;2:e000282. https://doi.org/10.1136/bmjph-2023-000282.

The funding statement in this article^1^ has been corrected to accurately reflect the nature of funding from the World Child Cancer - The Netherlands Foundation.

## What happened after publication

The authors and the World Child Cancer - The Netherlands Foundation contacted BMJ to request a correction, as the original funding statement incorrectly implied that the World Child Cancer - The Netherlands Foundation funded the study described in the article. The authors and the institution confirmed that the World Child Cancer - The Netherlands Foundation partly funded the institutional positions of co-authors Saskia Mostert and Minke Huibers, and did not directly fund the study.

## Reasons for correction

The funding statement has been corrected to reflect that the World Child Cancer - The Netherlands Foundation partly funded the institutional positions of Saskia Mostert and Minke Huibers, and did not directly fund the study.
